# The clinical use of stress echocardiography in ischemic heart disease

**DOI:** 10.1186/s12947-017-0099-2

**Published:** 2017-03-21

**Authors:** Rosa Sicari, Lauro Cortigiani

**Affiliations:** 1 CNR, Institute of Clinical Physiology, Via G. Moruzzi, 1, 56124 Pisa, Italy; 2 Department of Cardiology, San Luca Hospital, Lucca, Italy

## Abstract

Stress echocardiography is an established technique for the assessment of extent and severity of coronary artery disease. The combination of echocardiography with a physical, pharmacological or electrical stress allows to detect myocardial ischemia with an excellent accuracy. A transient worsening of regional function during stress is the hallmark of inducible ischemia. Stress echocardiography provides similar diagnostic and prognostic accuracy as radionuclide stress perfusion imaging or *magnetic* resonance, but at a substantially lower cost, without environmental impact, and with no biohazards for the patient and the physician.

The evidence on its clinical impact has been collected over 35 years, based on solid experimental, pathophysiological, technological and clinical foundations. There is the need to implement the combination of wall motion and coronary flow reserve, assessed in the left anterior descending artery, into a single test. The improvement of technology and in imaging quality will make this approach more and more feasible. The future issues in stress echo will be the possibility of obtaining quantitative information translating the current qualitative assessment of regional wall motion into a number. The next challenge for stress echocardiography is to overcome its main weaknesses: dependance on operator expertise, the lack of outcome data (a widesperad problem in clinical imaging) to document the improvement of patient outcomes. This paper summarizes the main indications for the clinical applications of stress echocardiography to ischemic heart disease.

## Background

### Pathophysiologic mechanisms

Stress echocardiography is the combination of 2D echocardiography with a physical, pharmacological, or electrical stress [1]. The diagnostic endpoint for the detection of myocardial ischaemia is the induction of a transient change in regional function during stress. A transient regional imbalance between oxygen demand and supply usually results in myocardial ischaemia, the signs and symptoms of which can be used as a diagnostic tool [1]. Myocardial ischaemia results in a typical ‘cascade’ of events in which the various markers are hierarchically ranked in a well-defined time sequence [2]. Flow heterogeneity, especially between the subendocardial and subepicardial perfusion, is the forerunner of ischaemia, followed by metabolic changes, alteration in regional mechanical function, and only at a later stage by electrocardiographic changes, and pain. The pathophysiological concept of the ischaemic cascade is translated clinically into a gradient of sensitivity of different available clinical markers of ischaemia, with chest pain being the least and regional malperfusion the most sensitive. The reduction of coronary flow reserve (CFR) is the common pathophysiological mechanism. Regardless of the stress used and the morphological substrate, ischaemia tends to propagate centrifugally with respect to the ventricular cavity: [3, 4] it involves primarily the subendocardial layer, whereas the subepicardial layer is affected only at a later stage if the ischaemia persists [4]. In fact, extravascular pressure is higher in the subendocardial than in the subepicardial layer; this provokes a higher metabolic demand (wall tension being among the main determinants of myocardial oxygen consumption) and an increased resistance to flow. In the absence of coronary artery disease (CAD), CFR can be reduced in microvascular disease (e.g. in syndrome X) or left ventricular (LV) hypertrophy (e.g. arterial hypertension). In this condition, angina with ST-segment depression can occur with regional perfusion changes, typically in the absence of any regional wall motion abnormalities during stress. Wall motion abnormalities are more specific than CFR and/or perfusion changes for the diagnosis of CAD [5–10].

## Diagnostic criteria

All stress echocardiographic diagnoses can be summarized into four equations centered on regional wall function and describing the fundamental response patterns: normal, ischemic, necrotic, and viable.

### Normal response

A segment is normokinetic at rest and normal or hyperkinetic during stress.

### Ischemic response

The function of a segment worsens during stress from normokinesia to hypokinesia (decrease of endocardial movement and systolic thickening), akinesia (absence of endocardial movement and systolic thickening), or dyskinesia (paradoxical outward movement and possible systolic thinning). However, a resting akinesia becoming dyskinesia during stress reflects purely passive phenomenon of increased intraventricular pressure developed by normally contracting walls and should not be considered a true active ischemia [1].

### Necrotic response

A segment with resting dysfunction remains fixed during stress.

### Viability response

A segment with resting dysfunction may show either a sustained improvement during stress indicating a non-jeopardized myocardium (stunned) or improve during early stress with subsequent deterioration at peak (biphasic response). The biphasic response is suggestive of viability and ischemia, with jeopardized myocardium fed by a critically coronary stenosis [1] Table 1.

**Table 1:** Stress echocardiography in 4 equations

Rest	+	Stress	=	Diagnosis
Normokinesis	+	Normo-Hyperkinesis	=	Normal
Normokinesis	+	Hypo, A, Dyskinesis	=	Ischaemia
Akinesis	+	Hypo, Normokinesis	=	Viable
A-, Dyskinesis	+	A-, Dyskinesis	=	Necrosis

## Ischaemic stressors

Exercise, dobutamine, and dipyridamole are the most frequently used stressors for echocardiographic test [11]. There are distinct advantages and disadvantages to exercise versus pharmacological stress. In Table 2 the stress echo protocols are reported.

**Table 2:** Stress echo protocols

Test	Equipment	Protocols
Exercise	Semi-supine bycicle ergometer	25 W x 2’ with incremental loading
Dobutamine	Infusion Pump	5 mcg/Kg/min 10-20-30-40 + atropine (0.25 x 4) up to 1 mg
Dipyridamole	Syringe	0.84 mg/Kg in 6’ or 0.84 mg/Kg in 10’ + atropine (0.25 x 4) up to 1 mg
Adenosine	Syringe	140 mcg/Kg/min in 6’
Pacing	External Pacing	From 100 bpm with increments of 10 beats/min up to target heart rate

### Exercise

Exercise echocardiography can be performed using either a treadmill or bicycle protocol. When a treadmill test is performed, post-exercise imaging is evaluated. Regional wall motion abnormalities would persist long enough into recovery to be detected but when recovery is rapid false-negative results occur [11]. Some Authors [12], perform peak-exercise imaging also during treadmill by keeping the patient standing still. Although challenging such an approach avoid false negative results in case of a rapid recovery to resting conditions. Information on exercise capacity, heart rate response, and rhythm and blood pressure changes are analysed and, together with wall motion analysis, become part of the final interpretation [11]. Bicycle exercise echocardiography is performed during either an upright or a recumbent posture. Unlike treadmill test, bicycle exercise allows to obtain images during the various levels of exercise. The supine position is the most suited for exercise echocardiography due to its ease of image acquisition in all views through the steps of the graded exercise [13]. In the upright posture, imaging is generally limited to apical views.

### Dobutamine

The standard dobutamine stress protocol consists of continuous intravenous infusion of dobutamine in 3 min increments, starting with 5 μg/kg/min and increasing to 10, 20, 30, and 40 μg/kg/min. If no endpoint is reached, atropine (up to 1 mg) is added to the 40 μg/kg/min dobutamine infusion [11, 14, 15].

### Dipyridamole

The standard dipyridamole protocol consists of an intravenous infusion of 0.84 mg/kg over 10 min, in two separate infusions: 0.56 mg/kg over 4 min, followed by 4 min of no dose and, if still negative, and additional 0.28 mg/kg over 2 min [11, 16, 17]. If no endpoint is reached, atropine (up to 1 mg) is added [18]. The same overall dose of 0.84 mg/kg can be given over 6 min [11, 19]. Aminophylline should be available for immediate use in case an adverse dipyridamole-related event occurs and routinely infused at the end of the test independent of the result [1, 11].

### Adenosine

Adenosine is usually infused at a maximum dose of 140 μg/kg/min over 6 min [3, 20]. When side-effects are intolerable, down-titration of the dose is also possible.

### Pacing

The presence of a permanent pacemaker can be exploited to conduct a pacing stress test in a totally non-invasive manner by externally programming the pacemaker to increasing frequencies. Pacing is started at 100 bpm and increased every 2 min by 10 bpm until the target heart rate or other standard endpoints are achieved [3, 21]. A limiting factor is, however, that several pacemakers cannot be programmed to the target heart rate.

## Indications to stress echo

Indications for stress echocardiography can be grouped in very broad categories which can encompass the majority of patients (see Table 3) [11]:

**Table 3:** Indications to stress echocardiography

Diagnosis of CAD in patients in whom exercise ECG is contraindicated, not feasible, uninterpretable, non-diagnostic or gives ambiguous results
Risk stratification in patients with established diagnosis
Pre-operative risk assessment (high-risk non emergent, poor exercise tolerance)
Evaluation after revascularization (not in the early post-procedure period, with change in symptoms)
Search for viability in patients with ischemic cardiomyopathy eligible for revascularization
Coronary artery disease of unclear significance at angiography or computed tomography

(i) CAD diagnosis;
(ii) prognosis and risk stratification in patients with established diagnosis (e.g. after myocardial infarction);
(iii) preoperative risk assessment;
(iv) evaluation for cardiac aetiology of exertional dyspnoea;
(v) evaluation after revascularization;
(vi) ischaemia localization;

As a rule, the less informative the exercise ECG test is, the stricter the indication for stress echocardiography will be. Out of five patients, one is unable to exercise, one exercises submaximally, and one exercises maximally but the ECG is uninterpretable.

*The three main specific indications for pharmacologic stress echocardiography* can be summarized as follows:

(i) patients in whom the exercise stress test is contraindicated (e.g. patients with severe arterial hypertension);
(ii) patients in whom the exercise stress test is not feasible (e.g. those with intermittent claudication);
(iii) patients in whom the exercise stress test was nondiagnostic or yielded ambiguous results: inability to achieve the target heart rate response, presence of chest pain in the absence of significant electrocardiographic changes, and a concomitance of conditions lowering the reliability of the ECG marker of ischemia (female gender, arterial hypertension, repolarization abnormalities on ECG under resting conditions or after hyperventilation, and the need to continue drugs such as digitalis or anti-arrhythmic that potentially induce ST-segment and T wave changes). Tables 4 and 5 report the main reasons for test interruption.

**Table 4:** Stress echocardiographic diagnostic criteria

Maximal dose or workload	
Target heart rate	
Echocardiographic Positivity (new or worsening of wall motion abnormality)	
Chest Pain	
ECG modification (ST Segment Shift > 2 mm)

**Table 5:** Submaximal non diagnostic criteria for test interruption

Intolerable symptoms	
Hypertension: Systolic Blood Pressure >220 mmHg; Diastolic Bllood Pressure > 120 mmHg	
Hypotension (Absolute or Relative): Blood Pressure Fall > 30 mmHg	
Supraventricular Arrhythmias: Tachycardia; Atrial Fibrillation	
Ventricular Arrhythmias: Ventricular Tachycardia; Polymorphous PVCs

2013 ESC guidelines on stable CAD have posed a new emphasis on the use of non-invasive imaging due to its significantly higher diagnostic accuracy [22]. However, the real impact of non-invasive imaging as a gate-keeper to coronary angiography need to to be tested on outcomes studies. The reported indications may change over time due to the evidence accumulating on survival impact. Exercise ECG remains a first-line in patients with interpretable ECG due to its simplicity, safety, availability, low cost, and high negative predictive value. Appropriateness criteria that may shed light on the use of stress echocardiography are derived from a balance between hard evidence, expert opinion, clinical experience and common sense.

## Diagnostic accuracy

In a meta-analysis of 55 studies with 3,714 patients, exercise, dobutamine, dipyridamole, and adenosine echocardiography showed a sensitivity, respectively, of 83, 81, 72, and 79%, and a specificity of 84, 84, 95, and 91% [23]. In another meta-analysis of 5 studies adopting state of the art protocols for dipyridamole (fast or atropine-potentiated) and dobutamine (atropine-potentiated) test, the two stresses had identical sensitivity (84%) and comparable specificity (92% vs 87%) [24]. When compared to standard exercise electrocardiography, stress echocardiography has a particularly impressive advantage in terms of specificity [25]. Compared to nuclear perfusion imaging, stress echocardiography at least has similar accuracy, with a moderate sensitivity gap that is more than balanced by a markedly higher specificity [23]. Familiarity with all forms of stress is an index of the quality of the echo lab. In this way, indications in the individual patient can be optimised, thereby avoiding the relative and absolute contraindications of each test. For instance, a patient with severe hypertension and/or a history of significant atrial or ventricular arrhythmias can more reasonably undergo to the dipyridamole stress test which, unlike dobutamine, has no arrhythmogenic or hypertensive effect. In contrast, a patient with severe conduction disturbances or advanced asthmatic disease should undergo the dobutamine stress test, since adenosine has a negative chronotropic and dromotropic effect, as well as a documented bronchoconstrictor activity. Patients either taking xanthine medication or under the effect of caffeine contained in drinks (tea, coffee, cola) should undergo the dobutamine test. Both dipyridamole and dobutamine have overall good tolerance and feasibility. The choice of one test over the other depends on patient characteristics, local drug cost and the physician’s preference. It is important for all stress echocardiography laboratories to become familiar with all stresses to achieve a flexible and versatile diagnostic approach that enables the best stress to be tailored to individual patient needs. Antianginal medical therapy (in particular, beta-blocking agents) significantly affects the diagnostic accuracy of all forms of stress; therefore, it is recommended, whenever possible, to withhold medical therapy at the time of testing to avoid a false-negative result [11, 26].

## Prognostic value

**Fig. 1:**
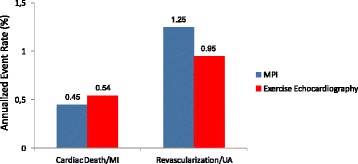
Annual cardiac events associated to a normal stress echocardiogram and a normal stress myocardial perfusion scan. From [43]

**Fig. 2:**
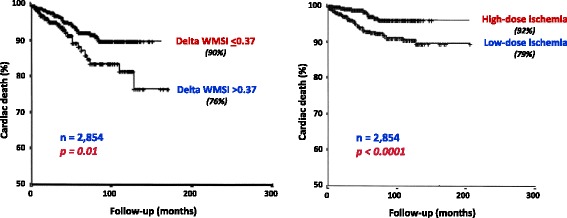
Kaplan-Meier survival curves (considering cardiac death as end point) in patients with pharmacologic stress echocardiography positive for ischemia stratified on the basis of the extent of ischemia, as expressed by delta wall motion score index (WMSI) set at 0.37 (*left panel*), and the dose to induce ischemia (*left panel*). From [47]

**Fig. 3:**
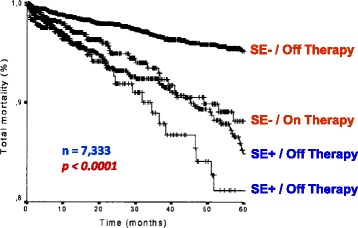
Kaplan-Meier survival curves (considering total mortality as end point) in patients stratified according to presence (DET +) or absence (DET -) of myocardial ischemia at pharmacological stress echocardiography on and off antianginal medical therapy. From [59]

The presence (or absence) of inducible wall motion abnormalities separates patients with different prognoses. Information has been obtained from data banks of thousands of patients - also with multicentre design - for exercise [27–43], dobutamine [44–52], and dipyridamole [25, 47, 53–57]. A normal stress echocardiogram yields an annual risk of 0.4–0.9% based on a total of >11000 patients [43], the same as for a normal stress myocardial perfusion scan (Fig. 1). Thus in patients with suspected CAD, a normal stress echocardiogram implies excellent prognosis and coronary angiography can safely be avoided. The positive and the negative response can be further stratified with interactions with clinical parameters (diabetes, renal dysfunction, and therapy at the time of test), resting echo (global LV function), and additive stress echo parameters (LV cavity dilatation, CFR, and previous revascularization). While the ischemic or necrotic pattern are associated with markedly increased risk of death or myocardial infarction, a normal test is predictive of a generally favorable outcome particularly in nondiabetic patients [58]. The ischemic response can be further stratified with additive stress echo parameters, such as the extent of inducible wall motion abnormalities and the workload/dose [47]. The higher the wall motion score index and the shorter the ischemia-free stress time are, the lower is the survival rate [47] (Fig. 2). As for the prognostic implication of the different pharmacological stress modalities, a similar prognostic value has been reported for dobutamine and dipyridamole testing [49]. Antiischemic therapy heavily modulates the prognostic impact of pharmacological stress echocardiography [59]. Inducible myocardial ischemia in patients on medical therapy identifies the subset of patients at highest risk of death. On the opposite end, the incidence of death in patients with a negative test off therapy is very low. At intermediate risk are those patients with a negative test on medical therapy or a positive test off medical therapy [59] (Fig. 3). The established prognostic stress echo parameters with their relative event rate are shown in Tables 6 and 7.

**Table 6:** Risk stratification for a positive test

1-year Risk (hard events)	Intermediate (1-3% year)	High (>10% year)
Dose/workload	High	Low
Resting EF	>50%	<40%
Anti-ischaemic therapy	Off	On
Coronary territory	LCx/RCA	LAD
Peak WMSI	Low	High
Recovery	Fast	Slow
Positivity or baseline dyssynergy	Homozonal	Heterozonal
CFR	>2.0	<2.0

**Table 7:** Risk stratification for a negative test

1-year Risk (hard events)	Very low (<0.5% year)	Low (1-3% year)
Stress	Maximal	Submaximal
Resting EF	>50%	<40%
Anti-ischaemic therapy	Off	On
CFR	>2.0	<2.0

## Stress echo in special subsets of patients

### Hypertensive patients

**Fig. 4:**
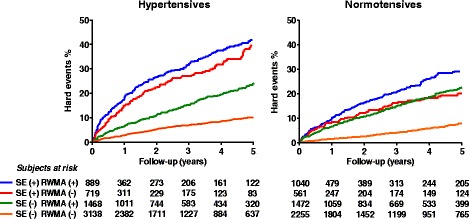
Hard event rates for hypertensive and normotensive patients separated on the basis of presence (+) or absence (−) of ischemia at stress echocardiography, and presence (+) or absence (−) of resting wall motion abnormality (RWMA). From [72]

In hypertensive patients CFR may be significantly reduced independent of the presence of significant CAD. CFR impairment reduces the diagnostic value of exercise electrocardiography and nuclear techniques in the hypertensive population, due to high rate of false-positive responses [10]. In hypertensive patients, stress echocardiography provides superior diagnostic specificity than exercise electrocardiography with no differences in sensitivity [60–62]. Moreover, dipyridamole stress echocardiography is most accurate than perfusion scintigraphy to assess coronary artery disease in patients with exercise electrocardiography positive for ischemia [63]. The prognostic assessment of hypertensive patients is of primary clinical importance since hypertension is associated with almost double risk of developing CAD [64]. Stress-induced wall motion abnormality during pharmacological testing is a strong multivariable predictor of future cardiac events in hypertensive patients with chest pain of unknown origin [65], including those with LV hypertrophy [66], and added prognostic information on top of clinical and exercise electrocardiography [67]. Moreover, the result of test is independently associated with cardiac death in an unselected cohort of hypertensive patients with known or suspected CAD [68]. However, mortality is predicted by inducible ischemia in exercise-tested patients and by the presence of any stress echocardiographic abnormality in those undergoing dobutamine challenge [69]. Inducible ischemia at inotropic stress is also associated with unfavourable outcome in the high-risk population of hypertensive patients unable to exercise [69]. Exercise-induced change in LV ejection fraction proved to be a multivariable predictor of mortality incremental to clinical findings, LV mass index, and resting LV function among hypertensive patients with LV hypertrophy [70]. Finally, echocardiographic LV wall motion abnormalities in adults without overt cardiovascular disease is associated with 2.4 to 3.4 fold higher risks of cardiovascular morbidity and mortality, independent of established risk factors [71]. It has been demonstrated in a large sample of 11.542 patients, that a normal study with any type of stressor is a marker of low risk; however in the hypertensive group the risk is higher [72]. Inducible ischemia at stress echocardiography is an independent predictor of hard cardiac events, and the level of risk is related to the extent of the inducible abnormality as expressed by peak wall motion score index [72] (Fig. 4).

### Diabetic patients

**Fig. 5:**
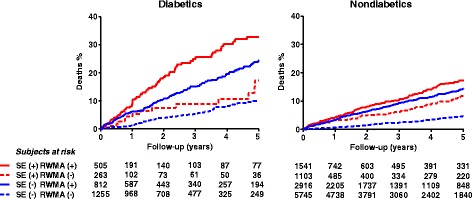
Mortality for diabetic and nondiabetic patients separated on the basis of presence (+) or absence (−) of ischemia at stress echocardiography, and presence (+) or absence (−) of resting wall motion abnormality (RWMA). From [85]

Exercise electrocardiography is of limited value in diabetic patients because exercise capacity is often impaired by peripheral vascular disease, neuropathic disease, and obesity. In addition, test specificity on electrocardiographic criteria is less than ideal due to high prevalence of hypertension and microvascular disease. Stress echocardiography can play a key role in the optimal identification of high risk diabetic patients, also minimizing the economic and biologic costs of diagnostic screening [1]. The coexistence of epicardial coronary artery stenosis with microangiopathy can explain the low specificity of perfusion imaging compared to stress echocardiography in the detection of CAD in asymptomatic and symptomatic diabetic patients [73, 74]. In diabetic patients, stress echocardiography has shown a higher specificity than perfusion imaging but suffers from higher rate of false-positive results, possibly due to the coexistence of cardiomyopathy in many patients [75]. Risk stratification of diabetic patients is a major objective for the clinical cardiologist, given their increased risk for coronary artery disease [76]. Several studies have addressed the prognostic ability of stratification of non-invasive imaging in patients with and without diabetes. In particular, in patients with overt resting ischemic cardiomyopathy, the presence of myocardial viability recognized by dobutamine stress echocardiography independently predicted improved outcome following revascularization in nondiabetics as well as in diabetic patients following revascularization [77]. Also in unselected patient populations with proven or suspected CAD, a clear refinement of prognosis can be obtained with stress echocardiography, first and foremost on the basis of classical wall motion abnormalities [58, 78–83], which place the patients in a high-risk subset for cardiovascular events. The incremental prognostic information provided by stress echocardiography is highest in patients with intermediate-to-high threshold positive exercise electrocardiography test results [84]. However, in diabetic patients – differently from nondiabetic subjects – a negative test result based solely on wall motion criteria is associated with less benign outcome [58] (Fig. 5). In a study on 2,349 diabetic patients investigated with dobutamine stress echocardiography, the mortality and cardiovascular morbidity were significantly higher in subjects with abnormal or ischemic test results [82]. Also, failure to achieve target heart rate and percentage of ischemic segments, an indicator of the extent of inducible ischemia, were independent predictors and incremental to clinical and rest echocardiographic variables for predicting adverse long-term outcomes [82]. In a more recent study assessing the long-term follow-up of stress echocardiography, dobutamine stress echocardiography provided restricted predictive value of adverse outcome in patients with diabetes who were unable to perform an adequate exercise stress test [83]. The Authors also identified a “warranty” period of the test which provided optimal risk stratification up to 7 years after initial testing. Repeated dobutamine stress echocardiography at that time might add to its prognostic value [83]. In a recent study enrolling a large sample of 14.000 diabetic and nondiabetic patients, stress echocardiography showed that a normal study with any type of stressor is a marker of low risk; however in the diabetic group the risk is higher [85] (Fig. 5). Inducible ischemia at stress echocardiography is an independent predictor of mortality, and the level of risk is related to the extent of the inducible abnormality as expressed by peak wall motion score index. However, the presence of rest wall motion abnormalities is an independent predictor of mortality in both patient groups [85]. Medical therapy at time of testing confers a higher risk of mortality in nondiabetics but did not appear to impact on outcome in the diabetic group [85]. The lack of modulation of stress testing by medical therapy suggest that inducible ischemia is more severe in the diabetic population. Stress myocardial perfusion imaging can be considered a viable alternative to stress echocardiography with the limitation of being less available, more expensive and with potential long-term downstream detrimental effect due to ionizing radiations [86]. Stress myocardial perfusion imaging has been shown to have significant prognostic power for future cardiac events in the symptomatic diabetic population. In a large multicenter study enrolling 4,755 patients (20% diabetic patients) who underwent exercise of pharmacologic myocardial perfusion imaging for symptomatic CAD, abnormal stress myocardial perfusion imaging was found to be an independent predictor of cardiac events in both diabetic and nondiabetic sample [87]. Moreover, the number of abnormal segments (fixed or ischemic) was related to worse outcome [87] and this is consistent with the stress echocardiographic findings showing an ominous outcome for higher values of wall motion score index.

### Women

**Fig. 6:**
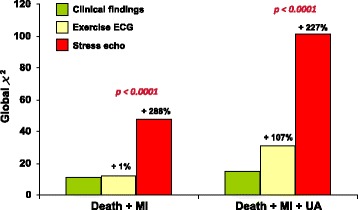
Incremental prognostic value of pharmacological stress echocardiography to clinical and exercise electrocardiography data, as determined by the comparison of the global chi-square at each step. From [93]

The diagnostic specificity of exercise electrocardiography and myocardial perfusion scintigraphy is definitely lower in women than in men. Reduction of coronary flow reserve in syndrome X (mostly affecting female patients), hormonal influences for exercise testing, and breast attenuation for nuclear technique are potential explanations. In contrast, echocardiography combined with exercise or pharmacologic agents provides similar sensitivity but a better specificity as compared to exercise electrocardiography [88, 89] and perfusion scintigraphy [90]. In women the prognostic value of stress echocardiography is high, similar to that in men [91]. In patients with chest pain of unknown origin, a normal test is associated with <1% event-rate at 3 years of follow-up, while an ischemic test is a strong and independent predictor of future events [92]. Moreover, stress-induced ischemia adds prognostic information on top of clinical and exercise electrocardiography data [93] (Fig. 6). In contrast to ECG stress test and perfusion imaging, stress echocardiography is an “equal opportunity” test, with no difference in diagnostic and prognostic accuracy between males and females. When exercise electrocardiography gives positive or ambiguous results, stress echocardiography is warranted [94]. The choice of an imaging test in this setting should take into account the radiologic burden. Recommendations from the European Society of Cardiology suggest to use non-ionising imaging techniques especially in highly vulnerable subjects such as younger women [87].

### Left bundle branch block

The presence of left bundle branch block makes the electrocardiogram uninterpretable for ischemia and, therefore, a stress imaging is necessary. The abnormal sequence of LV activation determines increased diastolic extravascular resistance, with lower and slower diastolic coronary flow, accounting for the stress-induced defect often observed by perfusion imaging in patients with normal coronary arteries [95]. In spite of the difficulty posed by abnormal wall motion, stress echocardiography is the best diagnostic option in patients with left bundle branch block. It is more specific than perfusion imaging [95], and its sensitivity is good, albeit reduced in the left anterior descending territory in the presence of a dyskinetic septum in resting conditions [96]. Moreover, myocardial ischemia by pharmacologic stress echocardiography has a strong and independent power in the prediction of future hard events in left bundle branch block patients, providing a prognostic contribution that is incremental to that of clinical and resting echo findings in the group without previous myocardial infarction [97].

### Noncardiac vascular surgery

Perioperative ischemia is a frequent event in patients undergoing major noncardiac vascular or general surgery and coronary disease is known to be the leading cause of perioperative mortality and morbidity following vascular and general surgery [98, 99]. The diagnostic/therapeutic corollary of these considerations is that CAD – and therefore the perioperative risk – in these patients has to be identified in an effective way preoperatively. This is not feasible in an accurate way with either clinical scores (such as Detsky’s or Goldman’s score) or rest echocardiography, only. The updated ESC guidelines recommend an imaging stress testing before high-risk surgery in patients with more than two clinical risk factors and poor functional capacity (Class I, Evidence C) [98]. Pharmacological stress echocardiography has been proven to be an effective tool for risk stratification when compared to perfusion scintigraphy [100–112]. Its advantages are due to lower costs and the lack of ionising radiations and in this particular setting it is more feasible than exercise stress echo. The experience with either dipyridamole and dobutamine indicates that these tests have a very high and comparable negative predictive value (between 90 and 100%) [113]. A negative test result is associated with a very low incidence of cardiac events allowing a safe surgical procedure [113–115]. The positive predictive value is relatively low (between 25 and 45%): this means that the post-surgical probability of events is low. Stress echocardiography shows a comparable diagnostic and prognostic accuracy when compared to perfusion imaging. The risk stratification capability is high for perioperative events and also remains excellent for long-term follow-up [116, 117]. Other techniques such as CMR and CCTA are available but the evidence is too scant to be recommended. Moreover, costs and risks should be weighed in the model of stratification and high tech imaging techniques do not seem to be adequate for a large scale assessment. Stress testing should be used in the diagnostic algorithm only when its result might influence peri-operative management and outcome. To date, it appears reasonable to perform coronary revascularization before peripheral vascular surgery in the presence of a markedly positive result of stress echocardiography in which standard medical therapy appears insufficient to prevent a peri-operative cardiac event. A more conservative approach – with watchful cardiological surveillance coupled with pharmacological cardioprotection with β-blockers – can be adopted in patients with less severe ischemic responses during stress [99]. Concerns have been raised on the initiation of beta-blockers before surgery without titration (to avoid hypotension and bradychardia) and in low risk patients [118]. Interestingly, clearly inappropriate indications for preoperative risk stratification before noncardiac surgery (intermediate risk surgery in patients with good exercise capacity, and low risk surgery) account for 25% of all inappropriate testing in large-volume stress echocardiography laboratories [119–122], and therefore this field provides a key opportunity for quality improvement and targeted educational programs to achieve measurable improvements in results.

## Myocardial viability

The stress echo sign of myocardial viability is a stress-induced improvement of function during low levels of stress in a region that is abnormal at rest. By far, the widest experience is available with low-dose dobutamine stress echocardiography [123–125] the preferred stressor for assessing myocardial viability. However, it is also possible to assess the presence of myocardial viability using low-dose dipyridamole [126–128] or low-level exercise [129] or enoximone [130, 131].

In patients with dysfunctional but viable myocardium, regional function can be improved by the inotropic effect of low-dose (5–10 μg/kg/min.) dobutamine stress echocardiography [11]. Sensitivity and specificity of low-dose dobutamine test are, respectively, 86 and 90% for predicting spontaneous functional recovery after an acute myocardial infarction (stunning) [123], and 84 and 81% for predicting functional recovery following revascularization in patients with chronic CAD (hibernation) [124]. Compared to nuclear techniques, dobutamine stress echocardiography has lower sensitivity, but higher specificity, with similar overall accuracy regarding recovery of function [124, 125]. In quantitative terms, contractile reserve evidenced by a positive dobutamine requires at least 50% viable myocytes in a given segment, whereas scintigraphic methods also identify segments with less viable myocytes [132]. Minor levels of viability, characterized by scintigraphic positivity and dobutamine echocardiography negativity, are often unable to translate into functional recovery. This explains the different diagnostic performance of the two methods.

Observational studies have indeed suggested that patients with ischaemic LV dysfunction and a significant amount of viable myocardium (at least five segments or a wall motion score index >0.25) [133–142] have lower perioperative mortality, greater improvements in regional and global LV function, fewer heart failure symptoms, and improved long-term survival after revascularization than patients with large areas of non-viable myocardium. On the other hand, viability has no impact on survival in patients with preserved or just moderately depressed LV function; in this case, it can rather predict the occurrence of acute coronary events, representing a substrate for unstable ischemic episodes [50].

Viability at dobutamine stress echocardiography predicts an improved outcome following revascularization both in diabetic and nondiabetic patients with ischemic cardiomyopathy [77]. No measurable performance difference for predicting revascularization benefit between stress echocardiography and nuclear methods has been reported [141]. The documentation of viable myocardium at dobutamine test also predicts responders to resynchronization therapy: patients with contractile reserve show a favourable clinical and reverse LV remodelling response to resynchronization therapy [143, 144].

## Coronary flow reserve

**Fig. 7:**
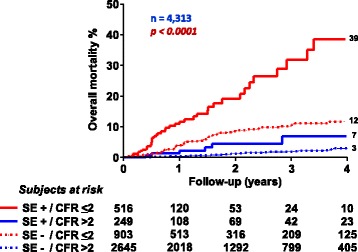
Mortality rates in patients with known or suspected coronary artery disease separated on the basis of presence (+) or absence (−) of ischemia at stress echocardiography (SE) and coronary flow reserve (CFR) of *left* anterior descending artery >2 or ≤2. From [145]

**Fig. 8:**
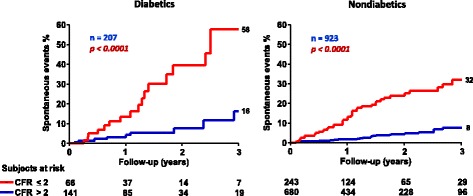
Hard event rates in diabetic and nondiabetic patients with stress echo negative for ischemia separated on the basis of coronary flow reserve (CFR) of *left* anterior descending artery >2 or ≤2. From [146]

**Fig. 9:**
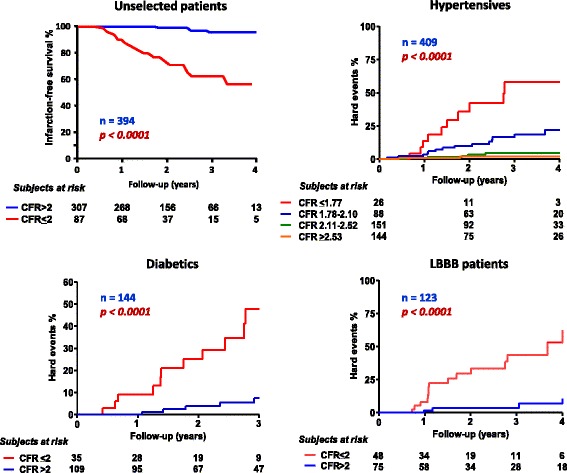
Hard event rates in unselected patients (*left upper panel*), hypertensives patients (*right upper panel*), diabetic patients (*left lower panel*), and patients with *left* bundle branch block (LBBB) (*right lower panel*) with normal or near normal coronary arteries separated on the basis of coronary flow reserve (CFR) values. From [147, 149–151]

**Fig. 10:**
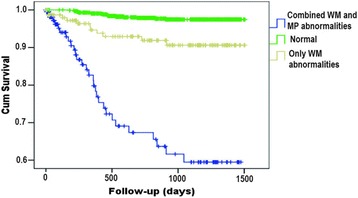
Kaplan-Meier curves based on the combination of presence or absence of wall motion (WM) abnormalities and myocardial perfusion (MP) abnormalities. From [152]

**Fig. 11:**
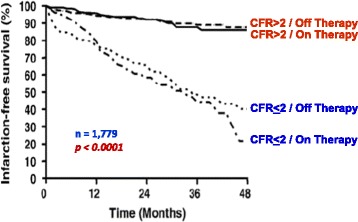
Kaplan-Meier survival curves (considering hard events as end point) in patients stratified on the basis of coronary flow reserve (CFR) of *left* anterior descending artery >2 or ≤2 on and off antianginal medical therapy. From [155]

In recent years the evaluation of CFR by combining transthoracic Doppler assessment of coronary flow velocities with vasodilator stress has entered the echo lab as an effective modality for both diagnostic and prognostic purposes [1]. The use of CFR as a stand-alone diagnostic criterion suffers from two main limitations. In fact, only left anterior descending artery is sampled with very high success rate. Moreover, CFR cannot distinguish between microvascular and macrovascular coronary disease [1]. Therefore it is much more interesting to assess the additional diagnostic value over conventional wall motion analysis. CFR of left anterior descending artery is a strong and independent indicator of mortality, conferring additional prognostic value over wall motion analysis in patients with known or suspected CAD [145] (Fig. 7). A negative result on stress echocardiography with a normal CFR confers an annual risk of death <1% [145] (Fig. 7). Moreover, CFR yields useful prognostic information in several clinical subsets, such as diabetics with unchanged wall motion during stress [146] (Fig. 8), hypertensives [147], patients with intermediate coronary stenosis [148], left bundle branch block [149], and normal or near normal coronary arteries [147, 149–151] (Fig. 9). A CFR <2.0 is an additional parameter of ischemia severity in the risk stratification of the stress echocardiographic response whereas patients with a negative test for wall motion criteria and CFR >2.0 during dipyridamole stress echocardiography have a favorable outcome. Similar results have been obtained when perfusion imaging was added to wall motion analysis [152] (Fig. 10). In diabetic patients, a normal CFR is associated with tighter glycemic control [153] and better long-term event-free survival both considering unselected patients [146, 154] and patients with angiographically normal coronary arteries [151] (Fig. 9). Within the subset of chest pain hypertensive patients with normal coronary arteries an effective risk stratification has been obtained according to quartilies of CFR [147] (Fig. 9). Anti-ischemic medication at the time of testing does not modulate the prognostic value of CFR, which is per se a prognostic marker independent of therapy [155] (Fig. 11). Specialist guidelines endorse an extensive application of CFR in the stress echo lab, suggesting that “whenever possible, it is recommended to perform dual imaging (flow and function) vasodilator stress echo [11].

## The shift from qualitative to quantitative reading

The search for a totally operator-independent quantitative tool for stress echocardiography is still on-going. The state-of-the art diagnosis of ischaemia in stress echocardiography remains the eyeballing interpretation of regional wall motion in black and white cine-loops. Several ultrasound technologies have been proposed in the last few years in order to overcome the qualitative interpretation of stress echocardiography: tissue characterization, Tissue Doppler imaging, strain rate and speckle tracking. However, none of these technologies has a place in the routine clinical practice of stress echo. EACVI/ASE Guidelines do not recommend its rotine clinical use unless some major limitations and pitfalls are solved [156]: “While the published research provides the evidence basis for potential clinical applications of these techniques in multiple clinical scenarios, the writing group believes that in the majority of areas, this methodology is not yet ready for routine clinical use. The consensus is that: (1) additional testing is needed in multicenter settings to better establish the diagnostic accuracy of the different parameters and their reproducibility in various disease states, (2) standardization is needed for what should be measured and how measurements should be performed, and (3) standardization among manufacturers is essential, as clinicians should be able to interpret data generated by different equipment irrespective of vendor”. It is conceivable that once the inter-vendor comparability is solved, speckle tracking would play a relevant role in determining forms of subtle LV dysfunction and minor degrees of subendocardial ischemia, otherwise non detectable with wall motion analysis. Real-time three dimensional echocardiography has also proven to be accurate and reproducible, but it remains time consuming and frame rates are too low for stress echocardiography [1]. The EACVI/ASE consensus document states that 3D stress transthoracic echocardiography holds promise for incorporation into clinical practice in the future [157]. Its advantages are: 1. Better visualization of the LV apex, which is frequently foreshortened on standard 2D apical images; 2. Rapid acquisition of peak stress images before the heart declines in recovery; and 3. Evaluation of multiple segments from different planes from a single data set. Disadvantages include lower spatial resolution and lower frame rates [157].

Myocardial contrast echocardiography (MCE) has undergone rapid development in the past 5 years. With the advent of newer-generation microbubbles, intravenous agents can now be used to both improve endocardial border delineation and, detect myocardial perfusion. Along with the development of microbubbles which consistently opacify the left heart from a venous injection, newer imaging modalities have evolved which permit the detection of myocardial perfusion abnormalities in real time, at frame rates which are greater than 25Hz. In order to accurately detect wall motion abnormalities during stress echocardiography, a clear definition of endocardial borders is of particularly importance. The approved indication for the use of contrast echocardiography currently lies in improving endocardial border delineation in patients in whom adequate imaging is difficult or suboptimal [158].

### Safety of pharmacologic stress echocardiography

Stress echocardiography is a safe technique but minor, limiting, side effects may preclude the achievement of maximal testing. Safety profile of stress echocardiography depends on the stressor used. Exercise is the safest stressor as shown in large samples with the long – lasting experience of ECG stress test [159]. Stress echo registries collecting data on thousands of patients [160–163] have shown that exercise is the safest test. Death has an incidence rate of 1 in 10,000 tests. Major life-threatening effects (myocardial infarction, ventricular fibrillation, sustained ventricular tachycardia, stroke) occur in about 1 in 6000 patients with exercise in the international stress echocardiography registry – fivefold less than with dipyridamole echocardiography and tenfold less than with dobutamine echocardiography. Exercise, whenever feasible, should be the preferred stressor due to its safety profile. Exercise is safer than pharmacological stress echocardiography and dipyridamole is safer than dobutamine . Not all stress tests carry the same risk of major adverse reactions and the choice of one test over the other should take into account the safety profile.

## Conclusions

Stress echocardiography is an established technique for the assessment of known or suspected CAD. It is recommended in all major cardiology guidelines in several clinical settings. However, its status of established technology, should prompt its clinical use as the preferred non-invasive imaging technique due to its low cost, wide availability and lack of radiation exposure. Though these unique features, an utilization gap remains with nuclear techniques percevied as more objective in the face of a comparable diagnostic and prognostic accuracy. The flexible use of stressors (exercise, inotropic and vasodilating) maximazes the feasibility, avoids specific contra-indications and allows tailoring the exam on each individual patient. A paradigm shift will occur when from a highly expertise qualitative reading stress echocardiography will move to a quantitative approach that would make it easier also for less skilled readers. Technological premises are there at hand but they have not reached a fullblown status to be used on a routine clinical basis. Society recommendantions and guidelines are mostly based on consensus and level of evidence C. The gap of knowledge should be filled with prospective large scale studies to support evidence-based treatment strategies. Eugenio Picano and Patricia Pellikka, two pioneers of the technique invite the scientific community to design “prospective large scale and (when possible) randomised (medical vs. interventional treatment) outcome studies to support more evidence-based rather than consensus-based strategies, based on stress echo results, in CAD and non-CAD patients” [164].

## Abbreviations

ASE: American Society of Echocardiography
CAD: Coronary artery disease
CFR: Coronary flow reserve
EACVI: European Association of Cardiovascular Imaging
ESC: European Society of Cardiology
LV: Left ventricle
